# Machine Learning and Machine Vision Accelerate 3D Printed Orodispersible Film Development

**DOI:** 10.3390/pharmaceutics13122187

**Published:** 2021-12-17

**Authors:** Colm S. O’Reilly, Moe Elbadawi, Neel Desai, Simon Gaisford, Abdul W. Basit, Mine Orlu

**Affiliations:** Department of Pharmaceutics, UCL School of Pharmacy, University College London, 29–39 Brunswick Square, London WC1N 1AX, UKm.elbadawi@ucl.ac.uk (M.E.); neel.desai.13@ucl.ac.uk (N.D.); s.gaisford@ucl.ac.uk (S.G.)

**Keywords:** artificial intelligence, industry 4.0, additive manufacturing, thin film manufacture, personalized pharmaceuticals, semi-solid extrusion (SSE), computer vision, drug-loaded systems, digital pharmaceutics & digital medicine, mobile 3D printing drug products

## Abstract

Orodispersible films (ODFs) are an attractive delivery system for a myriad of clinical applications and possess both large economical and clinical rewards. However, the manufacturing of ODFs does not adhere to contemporary paradigms of personalised, on-demand medicine, nor sustainable manufacturing. To address these shortcomings, both three-dimensional (3D) printing and machine learning (ML) were employed to provide on-demand manufacturing and quality control checks of ODFs. Direct ink writing (DIW) was able to fabricate complex ODF shapes, with thicknesses of less than 100 µm. ML algorithms were explored to classify the ODFs according to their active ingredient, by using their near-infrared (NIR) spectrums. A supervised model of linear discriminant analysis was found to provide 100% accuracy in classifying ODFs. A subsequent partial least square algorithm was applied to verify the dose, where a coefficient of determination of 0.96, 0.99 and 0.98 was obtained for ODFs of paracetamol, caffeine, and theophylline, respectively. Therefore, it was concluded that the combination of 3D printing, NIR and ML can result in a rapid production and verification of ODFs. Additionally, a machine vision tool was used to automate the in vitro testing. These collective digital technologies demonstrate the potential to automate the ODF workflow.

## 1. Introduction

Orodispersible films (ODFs) are an attractive delivery system, with clinically desirable features including high patient acceptability, requires no special administration instructions, and high drug loading [1,2]. For patients that have difficulty in swallowing, such as paediatric, geriatric and psychiatric patients, ODFs provide low risk of choking in comparison to conventional dosage forms [3]. Understandably, the market size is expected to reach USD 15.9 billion by the end of 2024, highlighting their economical and clinical rewards [4].

Despite the aforementioned benefits, both research and manufacturing of ODFs can be improved to enhance both clinical and manufacturing outcomes. For one, ODFs are batch manufactured, neglecting the current clinical paradigm of personalised medicines [5]. Conventional manufacturing methods of ODFs include solvent casting and film casting, where a bulk sheet of film is made and subsequently cut into the desired shapes. This post-fabrication process lacks sustainability for three reasons: (i) it requires a post-processing stage of cutting the films into size; (ii) materials are subsequently discarded, resulting in material wastage; and (iii) as the films are made in one bulk, more time is needed for drying. Hence, conventional casting techniques are time consuming processes and produce unnecessary material waste, which also makes them undesirable for clinical settings. Fortunately, these limitations can be addressed by three-dimensional (3D) printing. Three-dimensional printing is an emerging fabrication technology that has been recently demonstrated to produce bespoke, complex delivery systems with digital precision [6,7,8,9,10,11,12]. For ODFs, 3D printing can produce personalised dosage and only print the desired shape, resulting in faster drying times, no material wastage and considerably reduced manual labour in the post-fabrication stage [13,14,15,16,17,18,19]. Moreover, 3D printing is also an attractive candidate for on-demand medicine manufacturing, owing to their compact, versatile, and user-friendly attributes [20,21,22,23,24].

While 3D printing is more promising and autonomous than conventional ODF fabrication methods, there are several bottlenecks that need to be addressed before the technology is transferred to clinical settings. Despite its superior autonomy, there remains a trial-and-error approach to making delivery systems via 3D printing, which can be time-consuming. In addition, the prospect of transferring 3D printing into clinics can be improved by the introduction of real time release (RTR) testing. RTR testing is the ability to evaluate and ensure the quality of in-process and/or final product based on process data [25]—one example would be the combination of vibrational spectroscopy (e.g., near infrared (NIR) spectroscopy) and chemometrics as an alternative for conventional quality control [26,27]. NIR spectroscopy is a fast, non-destructive, operator-friendly, and portable method that could facilitate the identification of drugs and quantification of dose in dosage forms and can be integrated at the point of dispensing. Trenfield et al., (2020) demonstrated the potential of NIR spectroscopy as a non-destructive quality control method where they used a point-and-shoot approach to quantify the amount of paracetamol in printlets [28]. The final piece of the puzzle is the interpretation of the NIR spectra which is not something that would be feasible in clinical settings due to time constraints and lack of NIR spectroscopy expertise. Machine learning (ML) may provide the answer to this final barrier.

ML is a subset of Artificial Intelligence (AI) that builds predictive or decision-making models using historical data and improves upon these models through experience [29,30,31,32]. Like 3DP, ML is impacting several sectors [33,34,35], such as outperforming clinicians in diagnosis in the medical sector [36] and streamlining the drug to market process in the pharmaceutical sector [37,38]. However, the concurrent use of ML in 3DP of pharmaceuticals is a largely untapped resource as a decision-making tool in formulation development. While recent research has investigated the use of ML to address the trial-and-error approach of 3D printing [39,40], the RTR workflow remains underexplored.

The present study demonstrates the successful integration of both 3D printing and ML to automate the ODF fabrication workflow. To rapidly produce personalised ODF shapes, 3D printing was employed. Thereafter, the ODF prints were verified for both drug and dose using a combination of NIR and ML. The model drugs selected for this study were paracetamol, caffeine, and theophylline. The latter two are structurally similar, and hence were used to ‘stress test’ the ML models. Lastly, an additional digital technology, machine vision (MV), was also employed to analyse the in vitro disintegration of films. MV is also a subset of AI that captures visual information via a camera and converts it into digital information to be processed. The study discusses the potential of all three digital technologies to automate the research workflow and help facilitate the transition of 3D printing into clinical settings.

## 2. Materials and Methods

### 2.1. Materials

Blanose Carboxymethyl Cellulose Type 7HF-PH (725 kDa) was provided by Ashland (Wilmington, DE, USA). Acetaminophen (paracetamol) powder (A5000-1KG), caffeine powder (W222402-1KG-K), theophylline powder (T1633-1KG), and Patent Blue V sodium salt for microscopy (21605-25G) were purchased from Sigma Aldrich (Gillingham, UK).

### 2.2. Methods

#### 2.2.1. Feedstock Formulation

CMC feedstock formulations were prepared by adding the required amount of CMC slowly to 100 mL of deionised water in a beaker while stirring with a spatula at room temperature. The dispersion was left to stir for an hour on a hotplate stirrer with a magnetic stirrer rod to allow complete dissolution of the polymer and to allow dispersal of air bubbles. All mixing was performed at ambient temperature. For oral cavity model (OCM) analysis, formulations were dyed to visualise film dissolution by adding approximately 3 mL of 0.2% *w*/*v* Patent Blue to 100 mL of formulation. For NIR and ML analysis, CMC formulations were loaded with API (paracetamol or caffeine or theophylline) by adding the required amount of API to deionised water and allowing complete dissolution of the drug and then adding the required amount of polymer. Amount of API to achieve a certain % *w/w* film was calculated by assuming all water would be removed by the drying process, as shown in Table 1.

#### 2.2.2. 3D Printing Process

**Figure 1:**
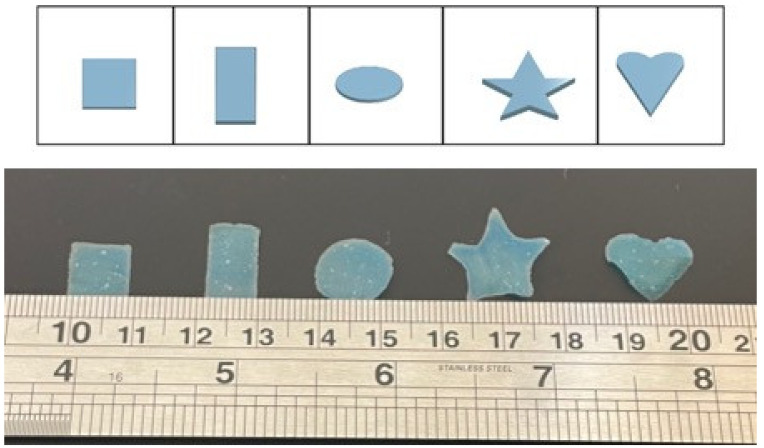
Examples of CAD designs of shapes (square, strip, circle, star, heart) used in this study (**top**) and examples of 3DP printed ODFs corresponding to the CAD designs (**bottom**) (Ruler markings in centimetres).

Onshape (Onshape Inc., Boston, MA, USA) was used to design the films used in this study. Figure 1 shows examples of each film design and image after printing, whereas representative images of drug-loaded films can be found in Figure S1. Designs were exported as stereolithography (.stl) files to the BioX Printer (Cellink, Gothenburg, Sweden), a direct-ink writing 3D printer, using a USB drive. Scotch Blue Trim and Baseboard’s Painter’s Tape #2093EL was applied to the petri dish prior to printing. Table 2 shows optimised printing parameters which were selected based on initial 3D printing process development.

Feedstock formulations were loaded into syringes then needle and compressed air line was attached to the syringe. Automatic bed levelling and calibration were performed to ensure uniform printing between batches. Once printed, films dried overnight at ambient conditions and then transferred to desiccator overnight to facilitate final drying. Thickness of films were measured on several locations on the films using a thickness gauge (Mercer Ltd., Manchester, UK). For OCM samples, 2.5 *w/v*% CMC were printed with a blue dye. For NIR and ML analysis, drug-loaded feedstocks from Table 1 were printed using the parameters in Table 2.

#### 2.2.3. Rheology

Rheology profiles of the feedstock formulations were gathered via rotational rheometry using a Bohlin Gemini HR Nano (Malvern Instruments, Malvern, UK) equipped with a 4°/40 Cone, with a gap of 150 μm. Samples were subjected to 25 different shear rates ascending logarithmically from 0.005 to 100 s^−1^ at 20 °C.

#### 2.2.4. Oral Cavity Model (OCM) Disintegration Testing

The OCM facilitates physiologically relevant disintegration testing by simulating the swallowing processes observed in the human mouth and was developed using information from various images of the human buccal cavity and other data [41]. Swallowing, and thus disintegration, is achieved through cyclic compression cycles of a silicone tongue against an acrylic ceiling, with simulated salivary fluid (SSF) introduced into the OCM from the anterior to posterior direction at a rate of 1.5 mL/min using a syringe driver [42].

The disintegration time was analysed using an MV algorithm. ODFs were placed in the middle of the tongue surface before starting the compression sequence. A mobile phone (Apple iPhone X, Apple Inc., Cupertino, CA, USA) was positioned on top of the acrylic plate with the camera facing down recording at 30 frames per second (fps). A single frame was extracted from the video between the compression and decompression phases and an area was calculated by identifying the perimeter of the ODF using MATLAB (MathWorks, Natick, MA, USA). For each ODF sample type tested mean and standard deviation of areas were calculated using built-in MATLAB functions (*n* = 3).

#### 2.2.5. Petri Dish Method Disintegration

ODF samples were placed in 90 mm petri dishes, which were positioned between springs of a water bath with shaker functionality (37 °C, 70 shakes/min). An amount of 2.5 mL of SSF was added directly onto samples in each petri dish. Disintegration times were recorded via stopwatch, and the point of disintegration established via operator observations (point at which structural integrity was lost). For each ODF type, *n* = 3 samples were tested.

#### 2.2.6. Near-Infrared Spectroscopy Analysis

The near infrared spectra were collected using a MPA Bruker FT-NIR spectrophotometer (Bruker, Global). Pure CMC, paracetamol, caffeine, and theophylline powder were used and were measured as references; *n* = 6 of each drug-loaded ODF type were scanned at a resolution of 1 cm^−1^ over a range of 12,000–4000 cm^−1^ for 64 scans, using a polytetrafluoroethylene (PTFE) backer.

#### 2.2.7. High-Performance Liquid Chromatography Analysis

High-performance liquid chromatography (HPLC; Agilent 1260 Infinity II, Agilent Technologies, Santa Clara, CA, USA) was used to verify drug dose. Calibration curve was generated covering the concentration range of the films, 0.01 to 2.5 mg/mL. The HPLC parameters for analysis of each drug varied and are enumerated in Table 3. An initial stock solution was prepared for each drug by pouring the drug into a 250 mL volumetric flask with the designated solution (Table 3). The stock solution was stirred using a magnetic stirrer until the powder completely dissolved. Working solutions were formed from the stock solution in 20 mL volumetric vials and diluted using the designated solution. For each solution, 50–60 µL was extracted using a syringe equipped with a 0.22 µm PTFE filter and subsequently poured into HPLC vials. The working solutions were used to generate the calibration curve. For analysing the drug-loaded films, the same protocol was followed. First, the weight of each film was recorded. Then, the films were placed in a 5 mL flask and subsequently mixed with the designated solution until complete film disintegration. Thereafter, the films were filtered and analysed via the HPLC.

#### 2.2.8. Machine Learning

ML models were developed using Python (version 3.9, Wilmington, DE, USA) to identify the drug and dose of API in the ODFs, using the scikit-learn package (version 0.17.1). Unsupervised learning using Principal component analysis (PCA) on the raw data was attempted to see if ML could inherently recognise the differences between groups without labelling. Subsequently, supervised learning using linear discriminant analysis (LDA) was applied to the data. LDA is a supervised alternative of PCA using labelled data which tries to maximise the distance between groups. For LDA, the data were first pre-processed to reduce signal noise using Savitzky-Golay filter (parameters: derivative = 0; window size = 21; and polynomial = 1st order), and then the model was trained using a training:testing split of 70:30 to see if the model could identify unseen data. The regression analysis was performed using partial least squares (PLS). A training:testing split of 75:25 was used, where a lower testing size was used to account for the smaller sample size used for regression analysis.

## 3. Results and Discussion

### 3.1. 3D Printing Method Development

**Figure 2:**
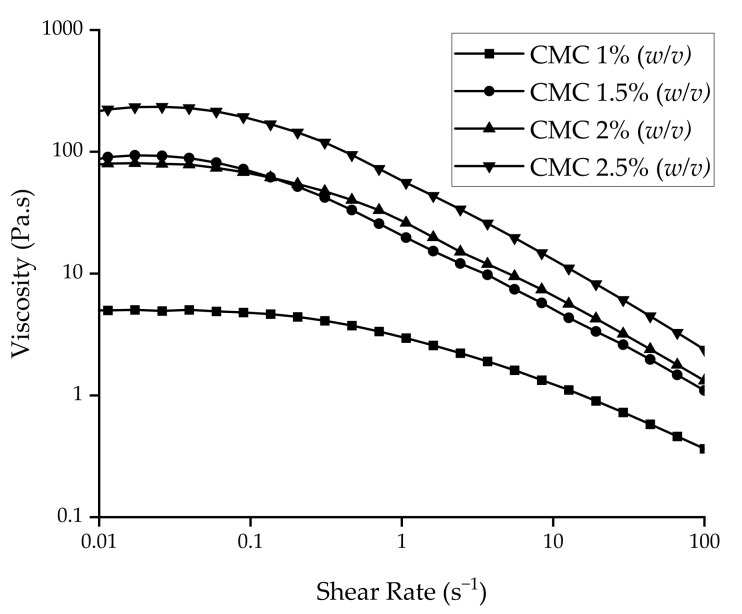
Rheology profiles of various concentrations of CMC feedstocks.

The present study sought to leverage digital technologies to automate, and in essence, simplify the ODF fabrication pipeline. CMC was chosen as the model polymer due to its pharmaceutical relevance. Several concentrations ranging from 1 to 2.5 *w/v*% CMC were printed. The ideal concentration for printing was 2.5 *w/v*%, which was found to both extrude and maintain print integrity post-extrusion. In contrast, 1 *w/v*% was found to extrude but subsequently spread, resulting in a distorted print shape. Rheological analysis (Figure 2) revealed that increasing the CMC concentration from 1 to 2.5 *w/v*% increased the Newtonian plateau by two orders of magnitude, from 10^0^ to 10^2^ Pa.s, which is in agreement with previous work [43]. Moreover, all formulations demonstrated shear-thinning properties, which is desirable in 3D printing [44,45]. Thus, it was deduced that the structural integrity of 2.5 *w/v*% was due to its higher viscosity values at low shear rates.

**Figure 3:**
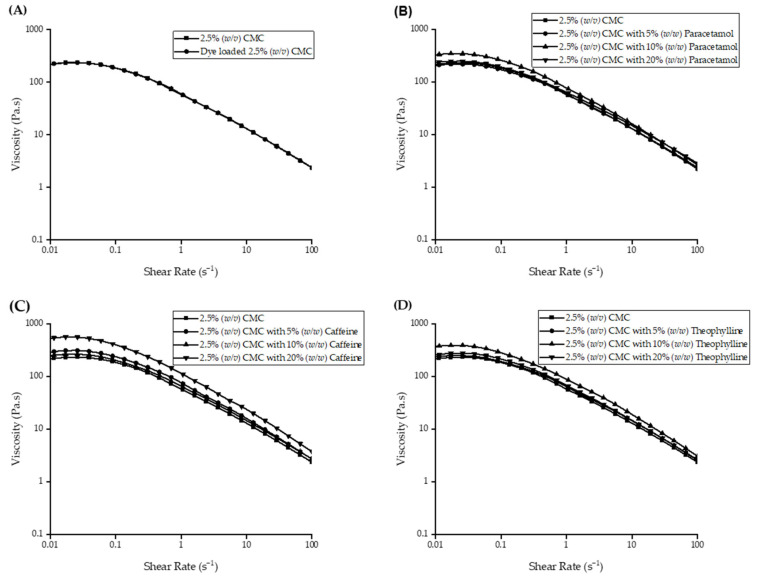
Rheological profiles of both (**A**) dye-loaded and (**B–D**) API-loaded 2.5 *w/v*% CMC solutions.

Further incorporation of materials, such as a dye for disintegration analysis or API, revealed minor increases in rheological properties to the selected CMC concentration. A dye was required for visualising the disintegration of films during OCM and MV analysis. The effects of the dye were negligible on the rheological profile, most likely due to the small amount of dye incorporated into the feedstocks, as illustrated in Figure 3A. The incorporation of APIs into 2.5 *w/v*% CMC concentration had minor effect on the viscosity (Figure 3B–D). The Newtonian plateau at low shear rates was found to remain in the order of 10^2^ Pa.s.

Based on these findings, formulations containing 2.5 *w/v*% CMC were used for subsequent analyses. The design and printing parameters were then optimised, where varying the infill density (ID) was found to affect the thickness of films. Strip designs resulted in film thicknesses of 73.34 ± 0, 95.56 ± 10.72 and 131.12 ± 9.63 μm, with ID of 25%, 40% and 50%, respectively. Thus, increasing the ID increased the film thickness. Strips with ID of 10% or below, and 50% or above were explored but these produced films with poor resolution. Hence, it was concluded that the ideal ID were 25% and 40%.

### 3.2. ODF Disintegration Study

**Figure 4:**
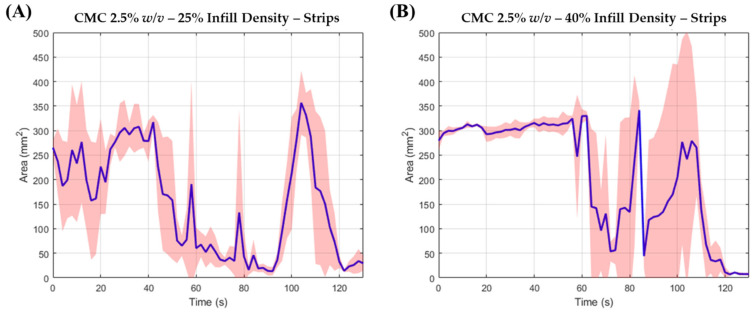
Mean top-down area-time profiles for (**A**) 25% and (**B**) 40% ID strip assessed in the OCM (*n* = 3).

**Figure 5:**
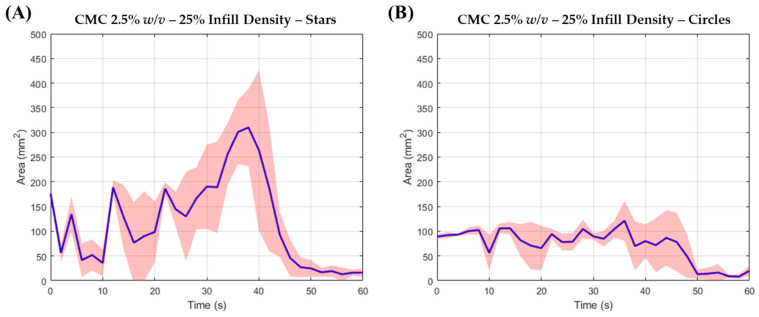
Mean top-down area-time profiles for (**A**) star and (**B**) circle geometries with 25% ID, assessed in the OCM (*n* = 3).

ODFs were assessed for their disintegration characteristics using the OCM. The data analysis process was automated via an MV algorithm. Strips with ID of 25% were found to disintegrate in under 180 s (Table 4) hence adhering to the Pharmacopeial requirement for suitable oral delivery [46], with a mean disintegration time of 80 s (Figure 4A). Strips with ID of 40% were less predictable when assessed using the OCM. These films remained intact for the first 90 s, and consistently fell off the tongue thereafter whilst remaining intact. Thus, it was difficult to ascertain whether ID of 40% would pass the European Pharmacopeial requirement of 180 s (Figure 4B). It was concluded that polymeric solutions of 2.5 *w/v*%, with 25% ID, was suitable for oral delivery, whereby films with good resolution were obtained, and were able to disintegrate within the European Pharmacopeial requirements. To further validate this observation, films of star and circle geometries were also examined using the OCM. Once more, it was discovered that these films were able to adhere to the European Pharmacopeial requirements (Figure 5). The thickness of star and circle shaped films printed with an ID of 25% were measured as 63.34 ± 5.78 μm and 93.34 ± 5.78 μm, respectively.

What is evident from both area-time profiles in Figure 4 and Figure 5 is a temporary increase in area compared to the area at *t* = 0 s. This increase reflects a temporary increase in blue dye over the synthetic tongue, as the film is being disintegrated, and subsequently releasing the blue dye. It is also evident that the temporary increase is followed by a catastrophic drop in area, reflecting the rapid removeable of the blue dye.

**Figure 6:**
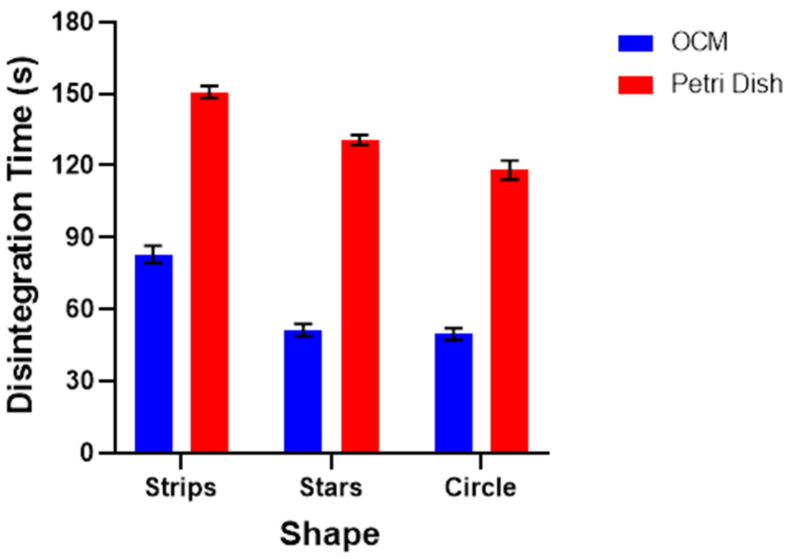
Disintegration time of films, as performed by both the OCM and modified petri dish.

A modified Petri dish disintegration study was performed to validate the results from the OCM disintegration studies. The observed disintegration times of strip (154 ± 8 s), star (131 ± 2 s), and circle (118 ± 4 s) geometries complied with Pharmacopeial requirements. As expected, the disintegration times were longer than that observed with the OCM, as the latter applied mechanical forces to accelerate disintegration (Figure 6). The modified petri dish results share a similar trend to the OCM observation, whereby both the star- and circle-shaped films presented with faster disintegration times. Further work is needed to explain this observation.

The above results demonstrated that DIW is a suitable technology for ODF fabrication. The ideal CMC solution was 2.5 *w/v*%, which was found to possess rheological properties to achieve ODF structures with suitable resolutions. Figure 1 demonstrates different shapes that can be generated with this CMC concentration. In addition, the layer height and optimal ID were 3 mm and 25%, respectively, which, post-drying, resulted in films with thickness below 100 µm. The film thicknesses reported herein are among the smallest values for 3D printed ODFs, such as those fabricated by fused deposition modelling (FDM), selective laser sintering and inkjet printing [28,47]. The advantage of DIW is that the process itself is rapid, and compared to FDM, feedstock preparation is rapid and does not require additional costly apparatus (i.e., hot-melt extruder). However, despite the aforementioned benefits, and the added advantages of rapid production and seamlessly producing different shapes, a limitation of DIW is the drying step. In this study, the films were left to dry overnight. This limitation affects other film fabrication technologies, including both solvent and film casting, but remains a subject of future work for the authors.

### 3.3. Drug and Dose Verification Using ML and NIR

A limitation of translating 3D printing to clinical settings that has been previously highlighted is quality control of the drug product produced [28]. Ideally this process should be accurate as well as automated. NIR spectroscopy is an industry-standard analytical method for quality control. It provides rapid measurements and can differentiate between different materials. However, the analytical process is performed manually. An emerging technology capable of automating both drug and dose verification from NIR spectroscopy is ML. There are several tasks that can be achieved by ML, of which classification and regression are pertinent to this study. Classification tasks aim to classify similar groups together by identifying patterns in the dataset, whereas regression tasks aim to predict continuous variables. In the context of this study, a classification task was first used to determine the predictive performance of ML to identify which ODF is being analysed. To ‘stress test’ the ML model, two drugs with similar chemical structures were purposefully selected (i.e., caffeine and theophylline). The similarity score of these drugs was 0.95, according DrugBank. This was then followed by a regression task to quantify the concentration of API in the film, as quantified by HPLC (Table S1, see Supplementary Materials). The benefit of drug verification can be for either the operator or for internet of things (IoT). For the operator, an ML pipeline that automatically identifies which drug to perform the dose verification for will mitigate human error. For IoT applications, a database of formulation parameters can be seamlessly recorded.

### 3.4. Classification of ODFs by API

Classification can be achieved by either supervised or unsupervised learning. Supervised ML requires the output to be labelled, such that the machine learning technique (MLT) knows the target it should predict. Unsupervised on the other hand allows the MLT to classify the samples without needing pre-labelled data, essentially asking the MLT to inherently detect patterns. The advantage of unsupervised learning is that it obviates the need for a user to pre-label the data, which in a dataset consisting of a large number of samples, can be time consuming. In this study, both supervised and unsupervised classification were explored to determine the optimal model.

**Figure 7:**
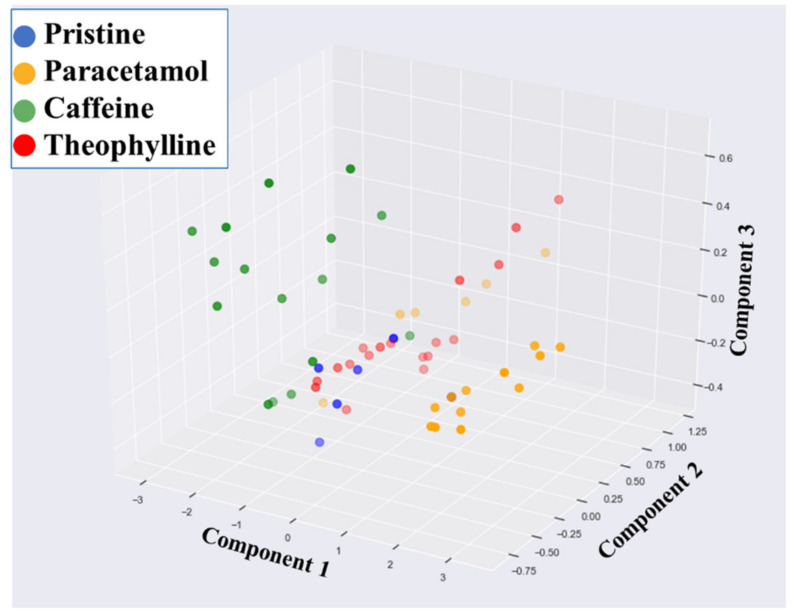
PCA decomposition of the raw NIR spectra to three dimensions (PCA 1—1st principal component 1; PCA 2—2nd principal component).

**Figure 8:**
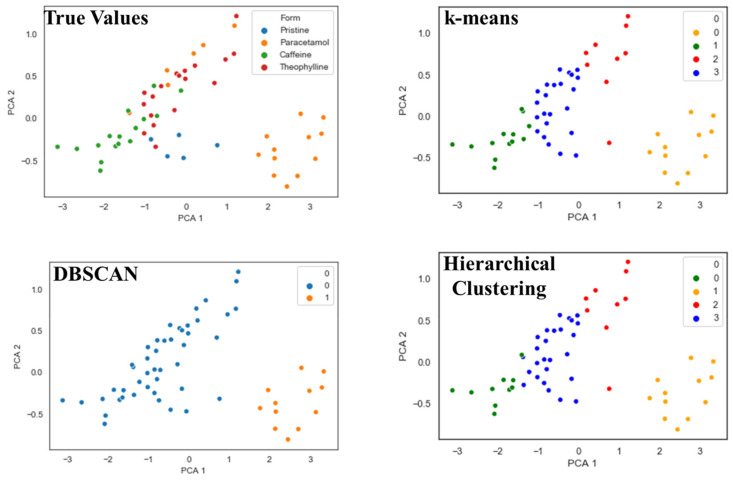
Two component PCA of the raw NIR spectra. PCA resulted in overlaps between clusters, which k-means, DBSCAN and hierarchical clustering were unable to exactly replicate. k-means and hierarchical clustering inherently identified four different clusters (clusters 0,1,2, and 3); however, they were not an exact match to the true values. DBSCAN was only able to detect two clusters (0 and 1) (PCA 1—1st principal component 1; PCA 2—2nd principal component).

Principal component analysis (PCA) is a common unsupervised learner with multiple applications in ML [48]. PCA was used to decompose the NIR spectra, consisting of 16,595 dimensions, to 3 dimensions that can be visualised. The NIR spectra were grouped by their API (i.e., paracetamol, caffeine, and theophylline) as well as the API-free films, which are referred to as pristine. The results of decomposing the NIR spectra using PCA are presented in Figure 7. PCA was able to approximate the different APIs into clusters to some extent. For example, paracetamol spectra and caffeine spectra were found to cluster to the right and left, respectively, in the three-dimensional space; thus, if a new ODF spectrum fell within either of these clusters then there is a high degree of certainty that it would contain the respective API. However, the clusters were found to overlap, and thus a 100% accuracy could not be achieved. This would pose a challenge if PCA was paired with a clustering algorithm which seeks to automatically detect clusters in datasets. To illustrate this hypothesis, three unsupervised clustering algorithms were used: k-means, density-based spatial clustering of applications with noise (DBSCAN) and hierarchical clustering. Figure 8 illustrates a two-component PCA plot, as well as the results of the three clustering algorithms. It can be observed that while all three clustering algorithms were able to successfully cluster the lower-right cluster (i.e., paracetamol), an exact match to the true clusters was not obtained for the other APIs.

**Figure 9:**
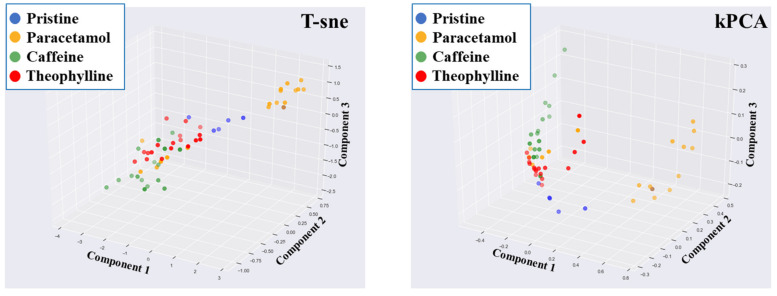
Non-linear transformation of the raw NIR spectra using T-sne and kPCA (kPCA1—1st principal component; kPCA2—2nd principal component).

In addition to PCA, t-distributed stochastic neighbour embedding (t-sne) and kernel PCA (kPCA) were also applied to the raw NIR spectra. One limitation with PCA is that it is a linear transformer, and hence assumes that the data possess a linear relationship. Both t-sne and kPCA are able to decompose the dataset using non-linear transformation. However, a similar finding to PCA was revealed, in that an exact clustering was not obtained (Figure 9). Therefore, it was concluded that PCA, t-sne and kPCA were unable to classify the APIs. 

**Figure 10:**
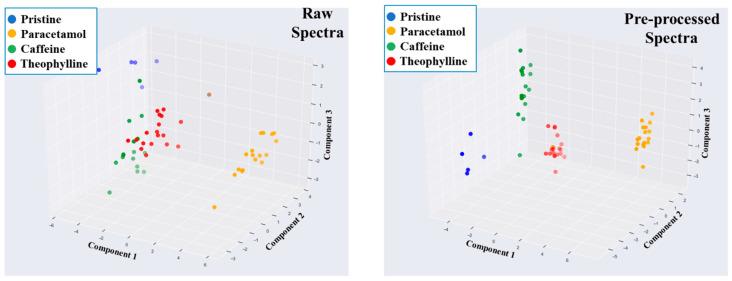
LDA classification of the raw and pre-processed spectra. Pre-processing of the NIR spectra yielded the highest accurate clustering.

**Figure 11:**
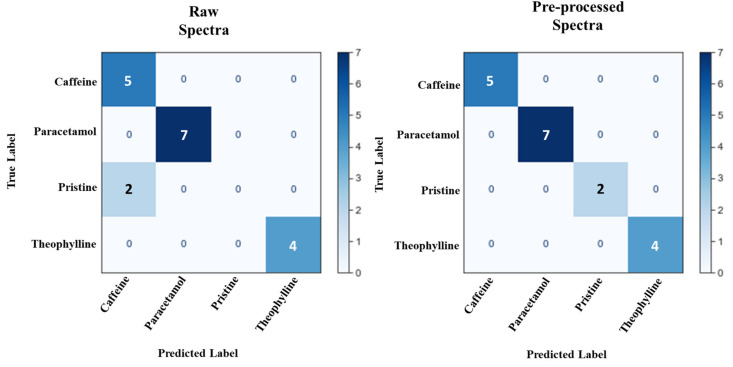
Confusion matrix depicting the results of the LDA prediction for both the raw and pre-processed NIR spectra.

As unsupervised learning was unsuccessful, supervised learning was then employed. A common supervised learner is linear discriminant analysis (LDA). This algorithm is similar to PCA; however, with the use of a pre-labelled dataset, it seeks to maximise the distance between the classes [49]. For this study, LDA was more effective in classifying the spectra according to their API constituent. Analysing the raw spectra using LDA yielded an improved separation between the different APIs (Figure 10) compared to the three unsupervised learners used. Unique clustering groups were observed for the paracetamol-containing films, with a minor overlap between the caffeine- and theophylline-containing films, as well as minor overlaps between the pristine and caffeine-containing films. A simple pre-processing step using a Savitzky–Golay filter resulted in complete separation between the different ODFs (Figure 10). Therefore, an ML pipeline of Savitzky-Golay pre-processing of NIR with LDA was found to be the most efficient pipeline for this study. To demonstrate the efficiency of this approach, the NIR dataset was split into 70:30, where the 70% was used to develop a predictive model (i.e., the training set), and the other 30% was used to evaluate the accuracy of the model (i.e., the testing set). A 100% prediction accuracy was achieved using the pre-processed spectra, whereas the accuracy with using the raw spectra was 88.9%. A confusion matrix of the result (Figure 11) revealed that two pristine spectra were predicted as caffeine, which is understandable as both were found to form clusters in similar data space.

### 3.5. Regression for Quantifying API Dose

**Figure 12:**
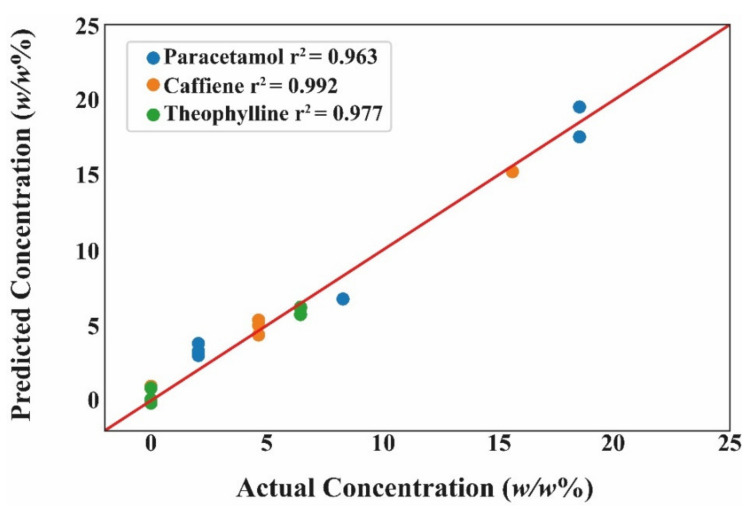
Comparing the predicted to the actual drug concentration on the test dataset for each API. The results are of the pre-processed NIR-ML data using partial least squares.

Following the successful clustering of films according to their API constituent, a regression model was developed to predict the API dose for each class. Partial least squares (PLS) was employed in this study. PLS is a common supervised learner suited for NIR owing to its ability to handle datasets where the number of dimensions are far greater than the sample size [50,51]. Applying PLS to the aforementioned ML pipeline, it was able to achieve an R^2^ value of above 0.99 for each of the classes. Each API class included the spectra of pristine as 0 *w/w*% API content. To test the accuracy of PLS, the dataset was split into training and testing, using a 75:25 split, which resulted in a predicted R^2^ of above 0.96 for each of the API class. Splitting the data into training and testing allows for the latter to essentially behave as ‘blind data’, which provides a more robust evaluation of the ML model (Figure 12).

### 3.6. Realising the Benefits of Digital Technologies

The study began by considering the tools needed to further enhance ODF development, with the aim of making the manufacturing process suitable for personalisation and on-demand production. It transpired that digital technologies possessed the tools needed to deliver this goal [52]. At the research setting, 3D printing, MV and ML were found to perform tasks whilst minimising human supervision. The former allowed multiple films to be produced once the process was started, whereas contemporary fabrication methods require each film to be manually cut. Similarly, MV was able to determine the disintegration times of films, which currently is performed through constant observation by the researcher. Lastly, ML was able to interpret NIR spectra, replacing the need for human interpretation of the individual peaks. It is also worth remarking that 3D printing and MV provided digital precision in the order of micrometres and milliseconds, respectively. ML on the other hand was found to rapidly complete the drug and dose verification task. The predictions made with the proposed ML pipeline took 0.091 s for LDA to be trained, and less than 0.001 s to classify one NIR spectrum. For PLS, the training and prediction per sample were 0.015 and 0.001 s, respectively. Moreover, in clinical settings, 3D printing and ML will ensure tasks can be replicated whilst diminishing the need for onsite expert supervision, which in turn will reduce expenses. The current work revealed that unsupervised algorithms were not 100% accurate in differentiating between the different APIs, particularly between caffeine and theophylline. Consequently, there will be a need for an expert to label the NIR spectra for training, which can be done offsite. Society, including both the pharmaceutics and healthcare sectors, is currently moving towards the next industrial revolution, Industry 4.0, where all three digital technologies are implicated therein [52]. The present study provides a strong validation for the use of digital technologies, showcasing their potential for an automated and reliable ODF workflow.

## 4. Conclusions

The present study confirmed that DIW was able to produce ODFs of bespoke shapes and with suitable disintegrating times. The ideal CMC concentration and infill were 2.5% *w/w* and 25%, respectively, which resulted in ODFs disintegrating far within the European Pharmacopeia specified 3 min. Three different API-containing films were fabricated (paracetamol, caffeine, and theophylline), where for each API category three varying concentrations of between 0–20% *w/w* were prepared. In addition, a robust ML pipeline was proposed for verifying both the drug and dose of ODFs. Both unsupervised and supervised learning were evaluated for drug verification, where it was discovered that supervised learning outperformed three different unsupervised learners. LDA was able to achieve drug verification accuracies of 88.9% and 100% with raw and pre-processed NIR spectra, respectively. Dose verification for all three API ODFs categories yielded high accuracies, with r^2^ values above 0.96. The study also revealed that the proposed ML pipeline was computationally undemanding, and suitable for scale-up with prediction times of 0.001 s per sample. Therefore, NIR spectroscopy and ML were confirmed to be suitable technologies for rapid drug and dose verification. Future work will concentrate in minimising the drying step of ODFs prepared by DIW.

## Figures and Tables

**Table 1:** Formulae for drug-loaded feedstock formulations.

Feedstock Formulation	Water (mL)	API (g)	CMC (g)
5% *w/w* Paracetamol	100	0.13	2.5
10% *w/w* Paracetamol	100	0.28	2.5
20% *w/w* Paracetamol	100	0.625	2.5
5% *w/w* Caffeine	100	0.13	2.5
10% *w/w* Caffeine	100	0.28	2.5
20% *w/w* Caffeine	100	0.625	2.5
5% *w/w* Theophylline	100	0.13	2.5
10% *w/w* Theophylline	100	0.28	2.5
20% *w/w* Theophylline	100	0.625	2.5

**Table 2:** Optimised printing parameters used in this study.

Parameters	
Needle gauge (Needle diameter)	22G (0.410 mm)
Compressed air pressure	100 kPa
Printing speed	20 mm/s
Infill pattern	Grid infill
Infill density	25%

**Table 3:** HPLC parameters.

Parameters	Paracetamol	Caffeine	Theophylline
Mobile phase composition	A: Distilled Water	A: Orthophosphoric Acid	A: Distilled Water
	B: Methanol	B: Acetonitrile	B: Acetonitrile
			C: Ethanol
Mobile Phase ratio	85:15	80:20	60:10:30
Flow rate (mL/min)	1	1	1
Injection volume (µL)	20	10	1
Detection wavelength (nm)	247	272	272
Column	Luna C18 (250 × 4.6 mm; 5 µm)	Luna C18 (250 × 4.6 mm; 5 µm)	Luna C18 (250 × 4.6 mm; 5 µm)
Column temperature (°C)	40	40	40
Retention time (mins)	8.8	13.0	13.3

**Table 4:** Extracted frames from videos showing ODF samples during disintegration in OCM (ID—infill density).

	Strip (25% ID)	Strip (40% ID)	Star (25% ID)	Circle (25% ID)
0 s	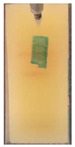	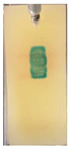	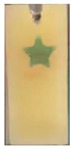	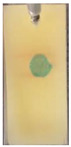
60 s	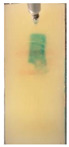	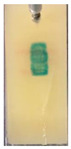	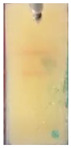	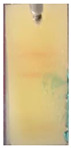
120 s	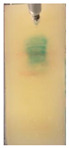	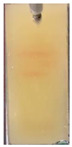	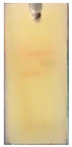	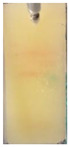
180 s	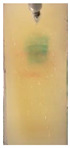	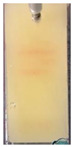	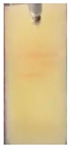	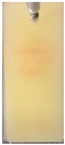

## Data Availability

The data presented in this study are available upon request from the corresponding author.

## Supplementary Materials

The following are available online at https://www.mdpi.com/article/10.3390/pharmaceutics13122187/s1, Figure S1: Representative low, medium and high-concentration drug-loaded film, Table S1: API concentration as determined by HPLC.
