# Supplementary material for: Machine Learning and Machine Vision Accelerate 3D Printed Orodispersible Film Development
Source: Pharmaceutics. 2021 Dec 17;13(12):2187. doi: 10.3390/pharmaceutics13122187 (PMC8706962; doi:10.3390/pharmaceutics13122187)
Supplement: Supplementary file 1 [file pharmaceutics-13-02187-s001.zip › pharmaceutics-1486056-supplementary.pdf]

pharmaceutics

Supplementary Materials: Combining Machine Learning and Machine Vision to Accelerate 3D Printed Drug-Loaded Orodispersible Films

Colm S. O'Reilly, Moe Elbadawi, Neel Desai, Simon Gaisford, Abdul W. Basit and Mine Orlu

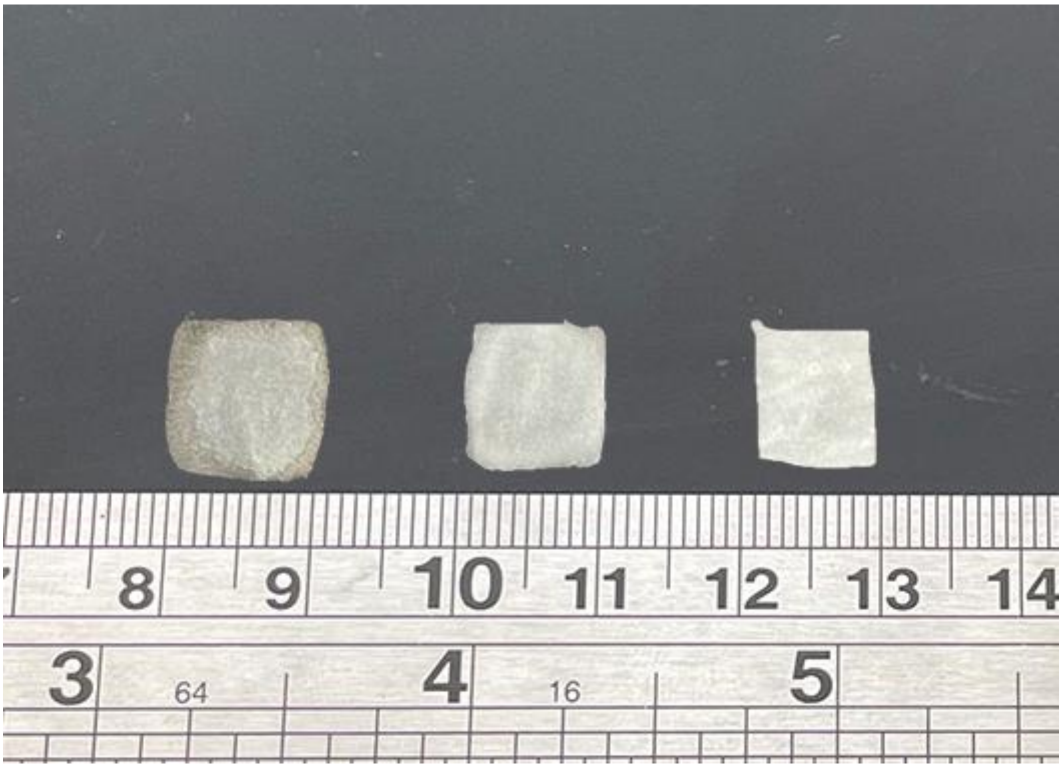

Figure S1. Representative low, medium and high-concentration drug-loaded film.

Table S1. API concentraiton as determined by HPLC.

Sample		API Concentration (*w*/*w*%)
Paracetamol	Low	2.05
	Medium	8.29
	High	18.51
Caffeine	Low	4.65
	Medium	7.28
	High	15.61
Theophylline	Low	3.29
	Medium	6.46
	High	14.72
